# The role of urinary N-acetyl-β-D-glucosaminidase in early detection of acute kidney injury among pediatric patients with neoplastic disorders in a retrospective study

**DOI:** 10.1186/s12887-022-03416-w

**Published:** 2022-07-20

**Authors:** Erika Bíró, István Szegedi, Csongor Kiss, Anna V. Oláh, Mark Dockrell, Robert G. Price, Tamás Szabó

**Affiliations:** 1 Department of Pediatrics, University of Debrecen, Faculty of Medicine, Nagyerdei krt 98, 4028 Debrecen, Hungary; 2 Department of Laboratory Medicine, University of Debrecen, Faculty of Medicine, Nagyerdei krt 98, 4028 Debrecen, Hungary; 3 SWT Institute for Renal Research, Epsom and St Helier University Hospitals NHS Trust, Wrythe Lane, Carshalton, SM5 1AA London, United Kingdom; 4 King’s College London, Stamford Street, SE1 9NH London, United Kingdom

**Keywords:** Tubular damage, Subclinical AKI, Cancer, GFR, Cystatin-C, uNAG

## Abstract

**Background:**

The 1-year cumulative incidence of AKI reportedly is high (52%) in pediatric neoplastic disorders. About half of these events occur within 2 weeks. However, subclinical AKI episodes may remain unrecognized by the conventional creatinine-based approaches. We investigated the diagnostic value of urinary N-acetyl-β-D-glucosaminidase (uNAG) as an early marker of acute kidney injury (AKI).

**Methods:**

In our retrospective study, 33 children with neoplastic disorders were inculded who had serial uNAG tests (at least 5 samples/patient) with a total of 367 uNAG measurements. Renal function was determined by cystatin-C and creatinine based GFR, and relative increase of uNAG index (uNAG_RI_). We focused on detecting both clinical and subclinical AKI episodes (according to Biomarker-Guided Risk Assessment using pRIFLE criteria and /or elevated uNAG levels) and the incidence of chronic kidney damage.

**Results:**

Sixty episodes in 26 patients, with positivity at least in one parameter of kidney panel, were identified during the observation period. We detected 18/60 clinical and 12/60 subclinical renal episodes. In 27/60 episodes only uNAG values was elevated with no therapeutic consequence at presentation. Two patients were detected with decreased initial creatinine levels with 3 „silent” AKI.

In 13 patients, modest elevation of uNAG persisted suggesting mild, reversible tubular damage, while chronic tubuloglomerular injury occurred in 5 patients.

Based on ROC analysis for the occurence of AKI, uNAGRI significantly indicated the presence of AKI, the sensitivity and specificity are higher than the changes of GFR_Creat_. Serial uNAG measurements are recommended for  the reduction of the great amount of false positive uNAG results, often due to overhydratation.

**Conclusion:**

Use of Biomarker-guided Risk Assessment for AKI identified 1.5 × more clinical and subclinical AKI episodes than with creatinine alone in our pediatric cancer patients. Based on the ROC curve for the occurence of AKI, uNAG_RI_ has relatively high sensitivity and specificity comparable to changes of GFR_CysC_. The advantage of serial uNAG measurements is to decrease the number of false positive results.

**Trial registration:**

The consent to participate is not applicable because it was not reqired for ethical approval and it is a retrospectiv study.

**Supplementary Information:**

The online version contains supplementary material available at 10.1186/s12887-022-03416-w.

## Introduction

The 1-year cumulative incidence of acute kidney injury (AKI) is reportedly high (52%) in pediatric patients with cancer, and roughly 50% of these episodes occur in the first two weeks after the initiation of anti-neoplastic treatments [1]. The potential spectrum of the outcomes of kidney injury includes AKI, chronic kidney disease (CKD), as well as many other disease conditions in between these endpoints; and the cumulative episodes of acute kidney injury increases the risk of CKD [2, 3]. Subclinical AKI, defined as elevation in levels of kidney damage biomarkers not fulfilling the conventional criteria for AKI, has characterized a subgroup of patients with increased risk of poor outcome [4].

The clinical diagnosis of AKI is based on parallel determination of serum creatinine (Creat_Se_), estimated GFR (GFR_Creat_) and urine output [5]. However, Creat_Se_ is neither sensitive nor specific enough [6], and may mask kidney problems in patient with malnutrition and decreased muscle mass, which conditions may be seen in patients with childhood cancer. Measuring cystatin-C (CysC) reportedly gives a more accurate result [7] and thus the diagnosis of AKI can possibly be established earlier than with Creat_Se_ [8].

Numerous studies have focused on the exact pathophysiology of AKI and its association with early tubular functional changes in the absence of morphological changes [9]. After gaining better understanding of the nature and dynamic changes of tubular biomarkers, the definition of AKI has broadened and according to Biomarker-Guided Risk Assessment other subgroups such as hemodynamic (biomarker negative and Creat_Se_ positive) and subclinical (biomarker positive and Creat_Se_ negative) forms of AKI have been created [10, 11]. That way recognition of AKI episodes became more effective, probably days before the elevation of Creat_Se_, and AKI-related morbidity can be more precisely predicted compared with using Creat_Se_ alone [12, 13].

Novel urinary markers for AKI (such as NGAL, KIM-1, uNAG) became available which opened a new era in the field of AKI detection methods [14].

The utility of uNAG as an indicator of the functional status of the renal tubules was described more than 40 years ago [15]. This enzyme originates from the proximal tubular epithelial cell lysosomes. NAG has a relatively high molecular weight, therefore it is not filtered through the glomerlural basal membrane and rapidly metabolized by the liver. Increase of urinary NAG usually indicates renal tubular cell breakdown, in parallel with kidney injury [16]. As previous publications highlighted, it is an excellent marker for follow-up or monitoring renal function during and after chemotherapy treatment in patients [17–20]. However, there has been some limitations reported with its use as an indicator of AKI, such as the uNAG activity has also been shown to be elevated in CKD patients, suggesting the lack of specificity for the purpose of risk stratification in AKI [19]. Moreover, uNAG was found to be elevated in other forms of active renal diseases, and in a variety of other conditions due to tubular injury, that could be associated with clinically significant AKI (rheumatoid arthritis, impaired glucose tolerance, hyperthyroidism, diabetes mellitus, nephrotic syndrome, urinary tract infection, perinatal asphyxia, heavy metal poisoning, several urological malformations) [16, 17, 21, 22].

The aim of our study was to analyze AKI episodes, including subclinical forms, in pediatric patients with neoplastic diseases using a urinary tubular marker uNAG; and to assess its diagnostic efficacy in early detection of AKI subtypes as compared with serum creatinine-based methods. We examined the proportion of tubular / tubuloglomerular damage following the observed discrepancy.

## Material and methods

### Study design

In our retrospective study we included children who had serial uNAG measurements (minimum 5 measurements/patient) between 2005–2019, treated in the Division of Hematology-Onkolgy of the Department of Pediatrics, University of Debrecen, Hungary.

Data were collected from the electronic database of the Clinical Center of the University of Debrecen (MedSol database). The time interval of uNAG measurements varied between 1–14 months, when the measurements were regularly, frequent and for a longer period of time monitored.

### Patients

Altogether, 33 patients (aged 1–16 years) were enrolled. Most patients presented with acute lymphoblastic leukemia, Wilms-tumor, lymphoma, or central nervous system tumor, while the remaining two children had Ewing-sarcoma and thymus tumor.

### Biological parameters

Frequency and timing of uNAG determinations were performed at the discreation of the pediatric hematologist-oncologist in charge. Before and during antineoplastic treatments, random and/or serial samples were taken based on the general condition of the patients with emphasis to nephrotoxicity due to possible infection or side effects of chemotherapy. Together with the kidney function (electrolites, Creat_Se_, etc.,) panel, samples for uNAG and CysC measurements were obtained. Sampling was done generally in the morning hours.

The analysis of the samples was performed at the Department of Laboratory Medicine, University of Debrecen, Hungary.

GFR values were calculated from creatinine and CysC values to properly compare laboratory parameters.

The GFR values based on creatinine (GFR_Creat_) values were calculated according to Full Ages Spectrum (FAS)-age GFR equation, which provide a good prediction value for patient without serious renal impairment [23, 24].

Likewise the CysC-based GFR calculation using Chronic Kidney Disease Epidemiology Collaboration GFR calculation with age 2012 (CKD-EPI) GFR Eq. (2012), which is an excellent and reliable way to assess renal function in pediatric patients [23, 25–27].

GFR calculation methods:

FAS GFR Calculation with Creat_Se_ values = $107.3/\lbrack{\mathrm{Creat}}_{\mathrm{Se}}\left(\mathrm{mg}/\mathrm{dL}\right)/{\mathrm Q}_{\mathrm{creat}}.$ 

$${\mathrm Q}_{\mathrm{creat}}=\;0.21\;+\;0.057\;\mathrm x\;\mathrm{age}\,-0.0075\;\mathrm x\;\mathrm{age}^2\;+\;0.00064\;\mathrm x\;\mathrm{age}^3-0.000016\;\mathrm x\;\mathrm{age}^4\;\mathrm{for}\;\mathrm{boys}$$

$${\mathrm Q}_{\mathrm{creat}}\;=\;0.23\;+\;0.034\;\mathrm x\;\mathrm{age}\;-\;0.0018\;\mathrm x\;\mathrm{age}^2\;+\;0.00017\;\mathrm x\;\mathrm{age}^3\;-\;0.0000051\;\mathrm x\;\mathrm{age}^4\;\mathrm{for}\;\mathrm{girls}$$

CKD - EPI GFR calculation with CysC values =

$$\mathrm{if}\;\mathrm{CysC}\;<\;0.8\;\mathrm{mg}/\mathrm L:\;133\;\times\;{(\mathrm{Scys}/0.8)}^{-0.499}\;\times\;0.996^{\mathrm{Age}}\;\lbrack\times\;0.932\;\mathrm{if}\;\mathrm{female}\rbrack$$

$$\mathrm{if}\;\mathrm{CysC}\;>\;0.8\;\mathrm{mg}/\mathrm L:\;133\;\times\;{(\mathrm{Scys}/0.8)}^{-1.328}\;\times\;0.996^{\mathrm{Age}}\;\lbrack\times\;0.932\;\mathrm{if}\;\mathrm{female}\rbrack$$

The uNAG was determined from urine samples by using a colorimetric assay, VRA-Glc-NAc assay. The uNAG index was calculated as the ratio of urinary NAG activity and urinary creatinine. The relative urinary NAG index (uNAG_RI_) means the elevation of uNAG index related to the upper limit of age-dependent uNAG index according to V. Oláh et al. [28, 29].

The uNAG_RI_ was considered to be positive for AKI detection when this value has reached at least 2 and in parallel the rise of uNAG_RI_ rate was minimum 1,5 compared to previous uNAG_RI_ of the same patient.

Recovery was defined as normalization of uNAG or a decrease of at least 80% compared to the highest value during the AKI event at that time.

### Nephrological classification

We focused on identifying clinical, subclinical AKI episodes according to the Biomarker-Guided Risk Assessment, as new nomenclature:

• hemodynamic AKI, as prerenal kidney injury: Creat_Se_ positive, tubular biomarker negative.
• subclinical AKI, indicates functional tubular disorder: tubular biomarker positive, Creat_Se_ negative.
• clinical AKI: tubular biomarkers, Creat_Se_ positive [10, 11].

In our study:

- hemodynamic AKI: Creat_Se_ positive, uNAG negative
- subclinical AKI: Creat_Se_ negative, uNAG and CysC positive:
  Due to avoid the incorrect conclusions from false-positive uNAGRI results.
- clinical AKI: uNAG, CysC and Creat_Se_ positive

For subclassification of clinical AKI the GFR-based system, the pRIFLE criteria was used for accurate comparison. The pRIFLE was developed using prospective data of critically ill children and it is a reliable method to detect the severity of AKI. Indeed, AKI defined by pRIFLE identifies some more patients with AKI (often refered to as R-AKI stages), than the KDIGO and AKIN systems, according to the compensative studies [30–32]. The categorization of severity of renal impairment we used the change in CysC-based GFR, because it has more accuracy and reliability comparing to GFR_Creat_ in high risk population, confirmed by previous studies as well [33–36].

pRIFLE-criteria, define three severity stages based on estimated GFR (eGFR) and urine output:

• Risk (R): the eGFR decreases by 25% and the urine output < 0.5 mL/kg/hr for 8 h.
• Injury (I): eGFR decreases by 50% and the urine output < 0.5 mL/kg/hr for 16 hours
• Failure (F): the eGFR decreases by 75% or the the eGFR < 35 ml/min/1.73 m2 and the urine output < 0.3 mL/kg/hr for 24 hours or anuric for 12 hours

### Data collection

In addition to the analysis of laboratory parameters, we collected data characterizing of the modality of the treatment (type of chemotherapy, antibiotic treatment, ACE-I therapy, dialysis), which may have contributed to the renal damage or modification of renal function.

General parameters of the patients are shown in Supplement Table 1 (column 1–5), the characterization of AKI episodes, detected as subtypes of AKI with the results of kidney panel can be seen in columns 6–8. Column 9 indicates the possible cause leading to AKI. In patients with persisting tubuloglomerular disorder, in further follow up period the monitoring of kidney function was done with FAS GFR_Creat_ (column 10).

Clinical data relating to urine output are not shown.

### Statistics

Statistical analyses were conducted using SPSS v.24.0.

However, this is a retrospective study; we calculated the optimal sample size based on calculation suggested cohort studies and it showed a minimum of 50 participants [37, 38]. Unfortunately, the investigated sample study size is only 33, however the calculation has a suggestive force on a retrospective cohort.

Correlation between GFR_Creat,_ uNAG_RI and_ GFR_CysC_ were calculated with the use of non-parametric Spearman correlation analysis, because the uNAG parameters did not show normal distribution according to Kolmogorov–Smirnov and Shapiro–Wilk tests.

The uNAG_RI_ values, and the daily GFR changes were analyzed along ROC curves, where a positive event had was defined as a minimum 25% decrease in GFR_CysC_ (according to pRIFLE), so in clinical and subclinical AKI, too.

The distribution of uNAG_RI_ data between the AKI and non-AKI subgroups was analyzed according to the Mann–Whitney test.

## Results

Upon the follow-up period all three listed parameters were sampled at the same time. During the 218 months follow-up period 60 episodes, with at least one renal marker positivity were recorded in 26 patients (Supplement Table 1). According to Biomarker-Guided Risk Assessment using pRIFLE criteria and/or elevated uNAG levels, the number of subclinical AKI episodes were 12/30, while clinical AKI episodes were present 18/30 cases. The most plausible explanation for these episodes is drug-related nephrotoxicity which is often seen after platinum-based chemotherapy or in sepsis-related kidney involvement. Both clinical and subclinical AKI episodes were most common in patients with leukemia. The incidence of total AKI events was 4,1% which was much lower than the literature.

We detected a high number of isolated uNAG_RI_ positivity (28/60), which was the most common in patients with central nervous system tumors.

The tubular damage almost completly recovered in 14/26 patients. The average time to normalization of uNAG_RI_ deviation 1.2 ± 1.05 month in mild injury, and was 2.8 ± 1.8 months in case of severe damage with major GFR_CysC_ deviation.

Among the patients, in whom retained tubular damage was detected (12/26), overlapping permanent tubuloglomerular abnormalities (5/26) were also seen. Prolonged damage of the tubular system was more common in patients with leukemia and central nervous system tumors as well than in cases with accompanied by multiple episodes of renal injury. We extended our follow-up in 5 patients and examined their kidney function by monitoring only the GFR_Creat_. Unfortunately, 3 patients were lost to follow up within one year (died or admitted to another hospital), full recovery seen in 1 patient while in 1 patient CKD-2 stage remained persistent.

Isolated reduction of GFR_CysC_ without parallel increase of uNAG_RI_ or decreased GFR_Creat_ was observed in two measurements in our series.

Our data was supplemented with non-parametric, Spearman’s correlation analysis, which showed that changes of the uNAG_RI_ and GFR_Creat_ statistically significant follow the changes in GFR_CysC_ (Table 1).

**Table 1:** Spearman’s correlation analysis of uNAG_RI_, GFR_Creat_ for the changes in GFR_CysC_

	**1: GFR**_**Creat**_	**2: GFR**_**CysC**_	**3: uNAG**_**RI**_
1	1.000		
2	0.565**	1.000	
3	-0.199**	-0.356**	1.000
Number of samples	359	363	367
Mean	117,78	101,90	3,10
SD	40,28	37,30	3,65

Significance: **p < 0,001

In the Sprearman correlation analysis, GFR_Creat_ and uNAG_RI_ variables proved to be significant for the changes in GFR_CysC_.

**Fig. 1:**
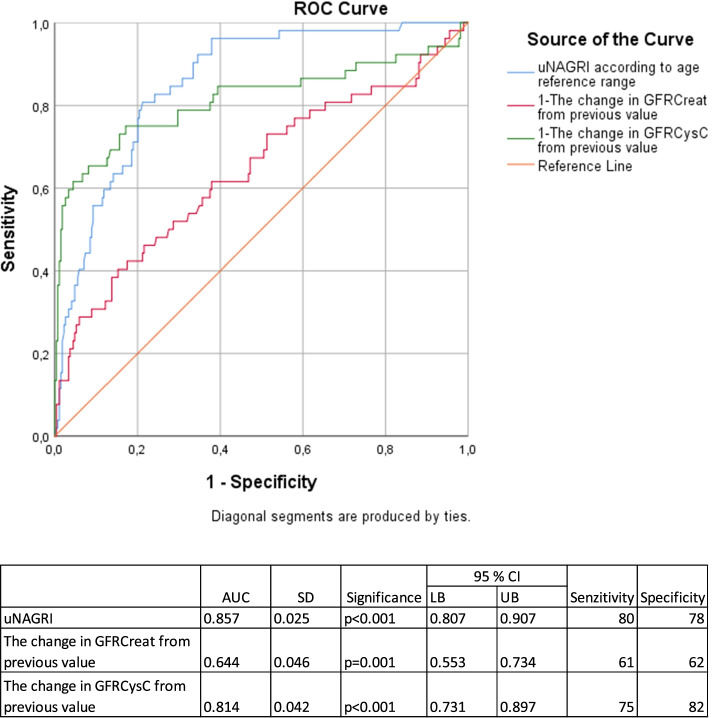
ROC analysis for the occurrence of AKI with the values of uNAG_RI_, the changes in GFR_Creat_ and GFR_CysC_ from previous values. ROC analysis for the occurrence of AKI, in which the uNAG_RI_ values were analyzed between the changes in GFR_Creat_ and GFR_CysC_ from previous values: uNAG_RI_ is usability to detect the AKI episodes

The Fig. 1 was to illustrate the usability of the uNAG method in the aspect of AKI recognition. The uNAG_RI_, as early AKI marker has overlapping precizity of GFR_CysC_ measurements and is superior to GFR_Creat_ measurement based on the analysis of the ROC curves (Fig. 1).

**Fig. 2:**
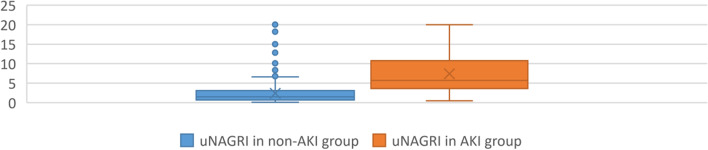
Distribution of uNAG_RI_ values between AKI and non-AKI groups using the non-parametric Mann–Whitney test. The groups differed significantly from each other (*p* < 0,001), but several outlayers were detected among non-AKI cases

In our analysis Mann–Whitney test of AKI and non-AKI uNAG_RI_ values were compared. It indicated significant difference between the AKI and non-AKI subgroups (including the total, clinical and subclinical AKI episodes) with a high number of outliers in the non-AKI group (Fig. 2).

## Discussion

The aim of our retrospective study was to explore in detail the subgroups (as clinical and subclinical AKI) according to Biomarker-guided Risk Assessment related in pediatric patients with neoplastic diseases, using a non-invasive, well-repeatable, low-cost tubular marker, the uNAG measurement.

The detection of AKI in pediatric oncology patients is difficult for several reasons. Major limitation of the creatinine-based detection is its latency (days later) to show the onset of AKI, because the Creat_Se_ value increases only when extensive glomerular/tubular involvement occurs. In addition, the detection of Creat_Se_ is affected by factors such as decreased muscle mass and malnutrition [39–41]. If the initial Creat_Se_ is low, the GFR_Creat_ is not sensitive enough to properly detect AKI. Indeed, we found decreased initial Creat_Se_ in 2 patients with 3 „silent” AKI episodes of which decreased creatinine level were recorded.

In our study we used the creatinine and CysC based GFR values for a proper comparability of GFR. Renal involvement and categorisation was examined according to the GFR based pRIFLE criteria.

Higher number of AKI episodes (1,5 x) were detected using tubular markers compared with only using GFR_Creat_. The diagnostic value of the uNAG_RI_ to detect AKI is also supported by our ROC curve. The number of AKI episodes tended to be higher in pediatric cancer patients who were on cisplatin-based protocols.

Similar observation was published by Bunnel et al. in a study where they used tubular biomarkers for the detection of AKI [17].

Nephrotoxicity seems to be more prominent in protocols using platina derivates, ifosfamide and cyclophosphamide [17–19, 42, 43], often complicated by the side effect of potentially nephrotoxic antimicrobial agents such as aminoglycosides, vancomycin, and amphotericin B [44]. Still, renal damage is often reversible in these cases requiring only closer monitoring, and optimal hydration [20, 45].

Hemodynamic AKI subtypes were not detected in our analysis, which is not surprising since pediatic oncology patients usually receive increased fluid intake (3000 ml/m2) to facilitate the elimination of chemotherapeutic agents and to reduce the chance of prerenal kidney involvement.

In our cohort we often detected isolated uNAG_RI_ elevation (27/60), which may be explained with increased volume intake when parallel decrease in urine creatinine level occurs. That alteration is reflected by mild/modest elevation of uNAGRI value. Repeated measurements of uNAG_RI_, may provide an accurate estimation of the actual tubular function.

Our data clearly showed that almost complete recovery (min 80% decreases of uNAG_RI_) of the tubular damage was observed in half of the cases, which was faster in cases without GFR_CysC_ deviation. In most cases with.

decreased GFR_CysC_, the glomerular function returned to normal after the normalization or significant decrease of uNAG, however, there was not conspicuous GFR_CysC_ improvement without uNAG decrease.

Early identification of AKI is important not only to avoid precipitating factors such as dehydratation, diuretic use or drug-induced nephrotoxicity but to prevent late consequences, such as permanent tubular dysfunction or CKD.

The incidence of CKD was reported to be higher in patients with a history of multiple AKI episodes [2, 3, 46].

In long-term survivors (follow-up > 5 years) severe renal disease (CKD grade ≥ 3) is quite rare, it only occurs in 0.5–0.8% of the patients [45]. According to the Renal Registry data, only 1.9% of renal failure seen in pediatric patients are associated with malignancies and on survivors of childhood cancer report that 0,5% had developed renal failure. However, their risk is significantly higher compared to their siblings (relative risk: 8.1) [47].

Permanent chronic damage was proved in our study only in one out of 35 cases which is a little higher compared with published data [45–47], may be due to low patients number.

Nowadays urinary NAG assays applicable on automated laboratory analysers are also available. More and more sensitive, reliable and cost-effective kits will be available that do not require a laboratory background once they have spread. Thus, as a non-invasive test can be a really easy-to-perform AKI pre-screening method [48–50].

There are certain limitations of our study such as the low number of patients and the individualized sampling pattern, which may have a distorting effect on the results.

## Conclusion

Our data demonstrated that precise assessment of actual kidney function required a more advanced methodology than a single creatinine measurement or creatinine-based GFR calculation.

Supplementation of the routine renal panel with an urinary tubular marker (like uNAG) may improves the detection of AKI. With its known limitations, repeated measurements of uNAG may provide significant help to detect and avoid nephrotoxicity by showing trends of changing in renal tubular damage. Even though our data strengthens previous observations, multicenter prospective studies with larger sample-size and long term follow-up are needed to draw further conclusion.

## Supplementary Information

**Additional file 1: Supplement Table 1.** Summary of AKI episodes and chronic kidney injury.

## Data Availability

Datasets analyzed during the study represent patient’s data available in their medical documentation and the electronic patients’ database (MedSolution) of the University of Debrecen for authorized personnel.

## Abbreviations

AKI: Acute kidney injury
CKD EPI GFR: Chronic Kidney Disease Epidemiology Collaboration GFR calculation with age 2012
CKD: Chronic kidney disease
Creat_Se_: Serum creatinine
CysC: Plasma cystatin-C concentration
FAS-age GFR: Full Age Spectrum equation eGFR
GFR_CysC_: Cystatin-C based CKD-EPI GFR
GFR_Crea_: Creatinine based CKD-EPI GFR
uNAG: Urinary N-acetyl-β-D-glucosaminidase
uNAG_RI_: Relative uNAG index (uNAG index/upper limit of age-dependent reference range)
