# Supplementary material for: The role of urinary N-acetyl-β-D-glucosaminidase in early detection of acute kidney injury among pediatric patients with neoplastic disorders in a retrospective study
Source: BMC Pediatr. 2022 Jul 20;22:429. doi: 10.1186/s12887-022-03416-w (PMC9297588; doi:10.1186/s12887-022-03416-w)
Supplement: Supplementary file 1 — Additional file 1: Supplement Table 1. Summary of AKI episodes and chronic kidney injury. [file 12887_2022_3416_MOESM1_ESM.docx]

Supplement Table 1: Summary of AKI episodes and chronic kidney injury

| **Tumor type** | **Age~~:~~**  **years**  **/sex:**  **F/M** | **Tumor-**  **Stage** | **Treat-ment Protocol** | **Follow-up time**  **(month)** | **Renal episode characterisation** | | | **Underlying condition resulting in AKI** | **Extra-follow up** |
| --- | --- | --- | --- | --- | --- | --- | --- | --- | --- |
|  |  |  |  |  | **Type** | **Changes of kidney markers** | |  |  |
|  |  |  |  |  | **Categorisation: based on pRIFLE- the changes of GFR_CysC_** | **uNAG_RI_ changes *** | **pRIFLE- the changes ofGFR _Creat_** |  | **CKD monitoring: only GFR_Creat_** |
| **Wilms tumor** | 3 /F | II/IR | SIOP 2001 WT post | 7 | GFR_CysC_ poz. | N** | Ø** | st.p. nephrect. |  |
|  | 6/M | III/IR +HR |  | 10 | uNAG_RI_ poz. | 600 % | N | st.p.nephrect, doxorub. |  |
|  | 4/M | III/IR |  | 3 | uNAG + GFR_Creat_ poz. | 250 % | R*** | st.p.nephrect.  doxorubicin |  |
|  |  |  |  |  | clinical: R | 300 % | R |  |  |
|  |  |  |  |  | uNAG_RI_ poz. | 150 % | N |  |  |
|  | 5/F | III/HR |  | 6 | uNAG_RI_ poz. | 150 % | N |  |  |
|  | 2/F | II/IR |  | 8 | subclinical | 150 % | ↑** | st.p. nephrect. |  |
|  |  |  |  |  | uNAG_RI_ poz. | 50 % | N |  |  |
| **Leukemia** | 12/F | B-ALL | ALL IC- BFM 2002 | 9 | clinical: I | 2900 % | I, | Aspergill. |  |
|  | 4/M | c-ALL |  | 13 | GFR_CysC_ poz. | N | N | HD-MTX | ↓( followed 4 years): CKD 2 |
|  |  |  |  |  | clinical:R | 800 % | R | CPM |  |
|  |  |  |  |  | clinical: R | 100 % | R | Aspergill. |  |
|  |  |  |  |  | uNAG_RI_ poz. | 100 % | Ø | ARSD, Asperill. |  |
|  |  |  |  |  | clinical: I | 60 % | N | Aspergill. |  |
|  |  |  |  |  | clinical: I | 100 % | I | st.p. nephrect., |  |
|  |  |  |  |  | clinical: I | 200 % | I | infection |  |
|  | 2/F | HR-ALL. |  | 9 | subclinical | 4900 % | ↑ | infection |  |
|  |  |  |  |  | subclinical | 1400 % | ↑ | sepsis, CFM |  |
|  |  |  |  |  | clinical:R | 150 % | N | sepsis |  |
|  |  |  |  |  | subclinical | 900 % | Ø | CPM |  |
|  | 4/M | c-ALL |  | 4 |  |  |  |  |  |
|  | 12/M | T-ALL |  | 7 | subclinical | 200 % | N | ACE, HDMTX |  |
|  |  |  |  |  | uNAG_RI_ poz. | 900 % | N | ACE, CPM, Inf. |  |
|  |  |  |  |  | subclinical | 200 % | N | MTX |  |
|  | 3/M | ALL |  | 14 | clinical: R | 400 % | I | CPM |  |
|  |  |  |  |  | clinical: R | 150 % | I | CPM, Aspergill. |  |
|  |  |  |  |  | clinical: I | 100 % | R | IFO+ Aspergill. |  |
|  |  |  |  |  | clinical: I | 1400 % | I | IFO+Aspergill. |  |
|  | 4/F | pre B-ALL |  | 4 | uNAG_RI_ poz. | 500 % | N | CPM |  |
|  | 8/M | r-ALL/2. | ALL-REZ BFM 2002 | 4 | subclinical | 130 % | N ֎ | MTX, IFO, donourubicin |  |
|  |  |  |  |  | uNAG_RI_ poz. | 100 % | N | MTX, IFO |  |
|  | 16/M | r-ALL/2. |  | 7 | subclinical | 200 % | N | MTX | ↑: followed 1 month, admitted other hospitals |
|  |  |  |  |  | uNAG_RI_ poz. | 200 % | N | ACE, MTX, Citarabin |  |
|  |  |  |  |  | subclinical | Ø | N | st.p. SC transpl. |  |
| **Lymphoma** | 8/M | HD, II/A . | GPOH-95 | 7 | uNAG_RI_ poz. | 1200 % | N | Rota infection |  |
|  | 15/F | HD, IV. |  | 3 | uNAG_RI_ poz. | 100 % | N ֎ |  |  |
|  | 15/F | HD, IV. | ABVD | 12 |  |  |  |  |  |
|  | 8/M | NHL | NHL-BFM 95 | 8 | clinical: I | 100% | I | st.p. HD, MTX |  |
|  | 14/M | T-NHL |  | 7 | clinical: F | 300 % | F**** | Aspergill. |  |
|  | 5/F | HD, IV. | BFM LL 2009 | 3 |  |  |  |  |  |
| **Central nervous system tumor** | 1/F | plx. choroid. tu. | Hunga-rian Brain Tumor Prot. | 3 |  |  |  |  |  |
|  | 12/F | astrocytoma |  | 3 |  |  |  |  |  |
|  | 10/F | medullo-  blastoma |  | 9 |  |  |  |  |  |
|  | 15/M |  |  | 1 |  |  |  |  |  |
|  | 16/F | neurofibro-  matosis |  | 3 | uNAG_RI_ poz. | 400 % | Ø | platina | ↓: tu.progressio  1 year later die |
|  |  |  |  |  | uNAG_RI_ poz. | 1700 % | N | platina |  |
|  |  |  |  |  | clinical: I | 450 % | R | platina |  |
|  | 5/M | neuroblast-  oma | RAPID COJEC | 6 | clinical: I | 500 % | I | platina | Normalisation: tu.progressio  1 year later die |
|  |  |  |  |  | subclinical | 200 % | N | platina |  |
|  |  |  |  |  | subclinical | 150 % | N | platina |  |
|  | 7/F |  | OJEC/  OPEC | 1 | uNAG_RI_ poz. | 200 % | Ø | platina |  |
|  | 4/M |  | SIOP Prot. | 12 | clinical: R | 2600 % | R | regitin | remission, kidney funct. recover |
|  |  |  |  |  | clinical: F | 600 % | F | platina, MTX |  |
|  | 16/M | inoperable brain tumor | Hunga-rian Brain Tumor Prot. | 7 | uNAG_RI_ poz. | 350 % | N | platina |  |
|  |  |  |  |  | uNAG_RI_ poz. | 200 % | N | platina |  |
|  |  |  |  |  | uNAG_RI_ poz. | 500 % | N | platina |  |
|  |  |  |  |  | subclinical | 150 % | N |  |  |
|  | 5/M |  |  | 12 | uNAG_RI_ poz. | 150 % | N | platina |  |
|  |  |  |  |  | uNAG_RI_ poz. | 300 % | Ø | platina |  |
|  |  |  |  |  | uNAG_RI_ poz. | 700 % | Ø | platina |  |
|  | 15/M | germinoma | BEP | 6 | uNAG_RI_ poz. | 130 % | Ø | platina |  |
|  |  |  |  |  | uNAG_RI_ poz. | 200 % | Ø | platina |  |
|  |  |  |  |  | uNAG_RI_ poz. | 140 % | Ø | platina |  |
|  |  |  |  |  | uNAG_RI_ poz. | 300 % | Ø | platina |  |
|  |  |  |  |  | uNAG_RI_ poz. | 350 % | Ø | platina |  |
| **Others** | 13/M | Ewing sarcoma | Euro 99 Ewing  APOC | 4 | uNAG_RI_ poz. | 500 % | N | sepsis |  |
|  | 15/F | thymus tu. |  | 1 | uNAG_RI_ poz. | 150 % | N | adriamicin |  |

Abbreviations and symbols:

*: the uNAG_RI_ values was pozitive, when the uNAG_RI_ was min.2 and the increase rate min 1,5 according to the patient previous / basal value. Here is the change visiable

**: N: normal level; Ø: not measured; ↑: increased level

***: RIFLE criteria: R: risk, I: injury, F: failure,

­­­ : That means, there is a persistant problem from the beginning to the end of the arrow, the values remain in the pathological range.

֎: the GFR is decreased at the beginning of observations period

st. p. nephr.: status postoperative nephrectomy

Aspergill.: Aspergillosis

ACE: ACE inhibitor therapy

In this summary table in addition to general patient data, we characterized the episodes of renal damages (according to pRIFLE criteria, and the new nomenclatura) during the observation period. Finally is analyzed, the causal factors, these abnormalities become chronic abnormalities or are able to regenerate. In the case of chronic abnormalities, we continued to monitor patients beyond the observation period, when renal function was monitored only by GFR_Creat_ alone.
